# Encouraging Physical Activity in Patients With Diabetes: Intervention Using a Reinforcement Learning System

**DOI:** 10.2196/jmir.7994

**Published:** 2017-10-10

**Authors:** Elad Yom-Tov, Guy Feraru, Mark Kozdoba, Shie Mannor, Moshe Tennenholtz, Irit Hochberg

**Affiliations:** ^1^ Microsoft Research Herzeliya Israel; ^2^ Technion - Israel Institute of Technology Faculty of Medicine Haifa Israel; ^3^ Technion - Israel Institute of Technology Faculty of Electrical Engineering Haifa Israel; ^4^ Technion - Israel Institute of Technology Faculty of Industrial Engineering Haifa Israel; ^5^ Rambam Healthcare Campus Institute of Endocrinology Haifa Israel

**Keywords:** reinforcement learning, physical activity, diabetes type 2

## Abstract

**Background:**

Regular physical activity is known to be beneficial for people with type 2 diabetes. Nevertheless, most of the people who have diabetes lead a sedentary lifestyle. Smartphones create new possibilities for helping people to adhere to their physical activity goals through continuous monitoring and communication, coupled with personalized feedback.

**Objective:**

The aim of this study was to help type 2 diabetes patients increase the level of their physical activity.

**Methods:**

We provided 27 sedentary type 2 diabetes patients with a smartphone-based pedometer and a personal plan for physical activity. Patients were sent short message service messages to encourage physical activity between once a day and once per week. Messages were personalized through a Reinforcement Learning algorithm so as to improve each participant’s compliance with the activity regimen. The algorithm was compared with a static policy for sending messages and weekly reminders.

**Results:**

Our results show that participants who received messages generated by the learning algorithm increased the amount of activity and pace of walking, whereas the control group patients did not. Patients assigned to the learning algorithm group experienced a superior reduction in blood glucose levels (glycated hemoglobin [HbA1c]) compared with control policies, and longer participation caused greater reductions in blood glucose levels. The learning algorithm improved gradually in predicting which messages would lead participants to exercise.

**Conclusions:**

Mobile phone apps coupled with a learning algorithm can improve adherence to exercise in diabetic patients. This algorithm can be used in large populations of diabetic patients to improve health and glycemic control. Our results can be expanded to other areas where computer-led health coaching of humans may have a positive impact. Summary of a part of this manuscript has been previously published as a letter in *Diabetes Care*, 2016.

## Introduction

Physical activity is highly recommended to patients with diabetes, as it is known that such activity leads to better control of glucose and other metabolic risk factors and improved quality of life [1]. Despite recommendations, most diabetic patients fail to perform regular physical activity [2]. A major objective of the caring medical team is to find better methods to encourage and incentivize physical activity in these patients.

Apart from the obvious aim of improving persuasiveness in the communication between the patient and the health care providers on the issue of exercise [3], there have been attempts to explore alternative approaches to improve adherence to physical activity in diabetic patients, including financial incentives [4] and community programs [5].

The smartphone revolution has brought entirely new opportunities for communicating with patients on a continuous basis and measuring movement, as well as other parameters, longitudinally.

A very large proportion (30%-70%) of the population in developed and developing countries owns a smartphone [6]. In the last decade, there have been multiple studies of mobile phone interventions using short message service (SMS) messages to improve health-related behaviors (reviewed in De Jongh et al [7]), and there are several previous studies that have tried to assess the effect of mobile phone apps in encouraging physical activity (reviewed in Bort-Roig et al [8]). These studies use random messages or a display that quantifies the amount of activity performed. None of these studies used a personalized learning algorithm to tailor messages to individuals. For example, two small-scale studies targeted patients with type 2 diabetes and took advantage of the ability of the patients’ smartphone to recognize physical activity patterns [9,10], but the feedback was either the count of the number of steps walked, with no encouragement message, or a feedback provided by the nurse that cannot be scaled to a wide audience.

The impact of wearable activity monitors (such as Fitbit, Apple iWatch, and Microsoft Band) on encouraging exercise has not been assessed yet in an academic research setting.

The novel means of persistent communication afforded by smartphones, coupled with the ability to provide reinforcement to patients, as well as the almost immediate means to quantify its effect, has a potential to improve patient care on a wide scale, but the use of personalized SMS messages that take into account the actual quantified behavior that needs to be reinforced has not been reported yet.

Machine learning algorithms aim to discover a pattern, usually from previously-collected data. Reinforcement Learning (RL) algorithms [11], in contrast, are algorithms that learn by observing the result of an action taken by them and so can be applied in settings where data are scarce or varying. RL algorithms have been successfully applied in areas ranging from computer games [12] to health [13]. In the latter, Paredes et al [14] used RL to select interventions to assist mildly depressed individuals, showing that RL-selected interventions were more effective than those selected using other strategies. Adaptive experimental design [15] has been used to speed clinical trials and optimize treatment in a hospital setting [16].

The idea of highly personalized interventions for medical research has been suggested in the past, mostly to evaluate individual interventions without temporal correlations. Nahum-Shani et al [17] proposed using just-in-time adaptive interventions (JITAIs), which “adapt over time to an individual’s time-varying status, with the goal to address the individual’s changing needs for support.” Whereas JITAIs were usually described in terms of predefined rules [18], Klasnja et al [19] suggested to implement them through microrandomized trials, where randomized interventions could be used to estimate the causal effect of interventions in JITAIs. In all these cases, interventions are commonly envisioned as a way to evaluate the effectiveness of single interventions. Here, we focus on learning a policy that will maximize physical activity when each person receives multiple interventions over time.

RL is frequently applied through algorithms, which assume that the states of the system and its environment can be deduced, such as Q-learning [20], or the ones that can also operate in a stateless environment such as temporal difference (TD, lambda) [12]. Both these algorithms can deal with discounted rewards in a principled manner. However, implementing these algorithms requires further assumptions (which can also be learned from the data) on the behavior of people, including, for example, the discount factor. In our implementation, described below, we chose a method which makes minimal assumptions on people’s behavior and the change in them over time. The aim of this study was to assess the effectiveness of automatically tailored, personalized feedback in increasing the adherence of diabetic patients to a personal physical activity regimen recommended by their diabetes specialist. We used a smartphone app that measured physical activity (especially walking) and sent feedback, in the form of SMS messages, to users. A learning algorithm, trained using the RL framework, was used to predict the message most likely to increase activity on the following day. The primary outcome of this study was persistent improvement in physical activity. The secondary outcome was improved glycemic control.

## Methods

### Overview

We developed a mobile phone app that runs in the background of patients’ smartphones and collects the amount of physical activity performed by patients. These data were transmitted to a central server.

Each morning an RL algorithm assessed, for each patient, which SMS message would likely increase the physical activity of the patient in the upcoming day, and that message was sent to the patient. Users were represented to the RL algorithm by their demographics, past activity, expected activity, and message history.

The effectiveness of each message was assessed the following morning by calculating the amount of activity the patient performed since the last message was sent to him or her, and this signal served as the reward for training the RL algorithm.

### Patient Characteristics

Adult patients with type 2 diabetes were recruited for a 26-week-long study from the endocrinology and diabetes outpatient clinic at a tertiary hospital. Inclusion criteria were nonoptimal glycemic control (glycated hemoglobin [HbA1c] over 6.5%), a sedentary lifestyle with no dedicated physical activity up to the recruitment to the study, and ownership of an Android-based smartphone with a data connection (Wi-Fi at home or cellular data). Exclusion criteria were other types of diabetes and any disability that precludes walking for 20 min. The study was approved by the institutional review board of Rambam Health Care Campus. All patients gave written informed consent. This trial was registered at ClinicalTrials.gov, registration number NCT02612402.

Note that HbA1c is the common measure for control of blood glucose level in people with diabetes. It refers to the levels of glycated hemoglobin, a form of hemoglobin that is measured to identify the average plasma glucose concentration.

At recruitment, all participants received information on the importance of physical activity and a personal prescription for an activity plan, including the number of sessions of activity per week and time span for each per session (ie, at least 2 hours of walking per week divided to 3 walking sessions per week). The target physical activity level was decided by the physician and the patient, taking into account the physical condition of the patient, medical disabilities, and significant schedule limitations.

A dedicated smartphone app was installed on the participant’s mobile phone. This app used the phone accelerometer to sense when participants were performing physical activity (defined as walking or running for 10 min or longer) and transmitted this information once every 2.5 hours to a central server. The app was verified for its ability to measure walking when the mobile phone was on the participant’s body, as well as in a bag or purse. Feedback was provided to patients through SMS messages.

To preserve battery life, the smartphone app sampled the accelerometer once every 3.5 min, and if walking was detected, kept monitoring the accelerometer until no walking was detected. Only contiguous walks of 10 min or more were collected, as shorter walks have a small effect on improvement in clinical outcomes. Patients were told at recruitment that only walks 10 min or longer will be counted and were asked to carry their cellphone during such walks.

Intensification of dietary or medical treatment was not restricted when this was considered appropriate by the medical team. The HbA1c measurements were performed by standard procedures before recruitment and every 3 months in the health maintenance organization lab of each subject. The patients filled a Quality of Life Questionnaire [17] before and after 6 months of participation in the study. They also filled a short questionnaire assessing satisfaction of the experience of using the app.

### Types of Feedback Messages

Patients were randomized into a control arm and a personalized arm. The medical team was blinded to the type of messages each subject received. The control arm received identical unchanging once-weekly reminders to exercise. Patients in the personalized arm received daily feedback messages and weekly summaries.

We note that there are differences in both frequency and content between the messages for the control and treatment arms. For this reason, as will be explained below, two policies were used for the treatment arms, and these are further compared.

Following Elliot and Church [18], we sought to have three types of messages: mastery, performance-approach, and performance-avoidance, as well as a no-message condition. The daily feedback messages could be one of the following four messages (in parenthesis, the nomenclature of [18]):

1. Negative feedback: “You need to exercise to reach your activity goals. Please remember to exercise tomorrow” (Performance avoidance).
2. Positive feedback relative to self (referred herein as positive-self): “You have so far achieved N% of your weekly activity goal. Your exercise level is in accordance with your plan. Keep up the good work” (Mastery).
3. Positive feedback relative to others (referred herein as positive-social): “You have so far achieved N% of your weekly activity goal. You are exercising more than the average person in your group. Keep up the good work” (Performance approach).
4. No message

The percentage of the weekly goal (“N%”) was given as an integer greater than or equal to zero, computed according to the length of activity so far, compared with the length of activity expected given the exercise plan of the individual.

In general, messages did not necessarily reflect reality. For example, patients were not divided into groups, as is implied in the positive-social message. Similarly, a negative message might be sent even though the patient has already achieved their activity goal. However, to allow the algorithm to learn a policy, we did not set constraints on the possible messages to be sent.

On most weeks, the weekly summary message was as follows: “Please remember to exercise this week to reach your activity goals.” When patients reached a significant exercise achievement (as described below), and not more than once per 3 weeks, they could receive one of the following messages:

1. Maximal increase: Over the past week you increased your activity more than at any previous week.
2. Significant increase: Over the past week you increased your activity more than most previous weeks.
3. Maximal social: You won the first place! Last week you increased your activity more than any other participant in the experiment.
4. Significant social: Last week you increased your activity more than most participants of the experiment.

SMS messages were not sent to participants whose data were not received 12 hours or more before the current time to reduce the chance that the system would send a message based on incorrect data.

### Feedback Message Policies

**Figure 1:**
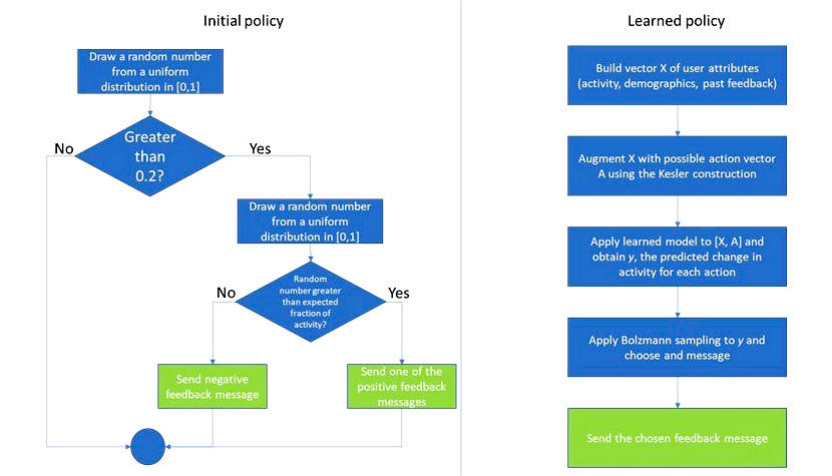
The two message policies.

After an initial period of 3 months where feedback was sent according to a predetermined policy detailed below (“initial policy”), the decision on which daily feedback message to send was decided by a learning algorithm (“learned policy”). To allow the algorithm to collect information of outcomes to less likely feedback policies, exploration [19] was used for messages that were deemed less likely to succeed such that they were sent with significant probabilities, as detailed below. Figure 1 shows an outline of the two feedback policies.

The initial algorithm (herewith referred to as “Initial policy”) was set so that no message was sent on 20% of days. For the remaining days, we drew a uniform random number between 0 and 1. If that number was larger than the expected fraction of weekly activity on that day, the user would receive the negative feedback message. Otherwise, they would receive one of the positive messages, with equal probability. This policy was based on the results of Elliot and Church [18] but also provided sufficient randomness for exploration.

After a sufficient number of instances were collected, we implemented a learned decision mechanism for deciding on the feedback message. This mechanism received, for each user, the following set of attributes:

1. Activity attributes:
  - Number of minutes of activity in the last day.
  - Cumulative number of minutes of activity this week.
  - Fraction of activity goal.
  - Fraction versus expected at this point in the week.
2. Demographics:
  - Age
  - Gender
3. Feedback attributes: Number of days since each feedback message was sent.

The attributes allowed the algorithm to model each person on each day through several aspects relevant to their behavior, including past activity (through the activity attributes), their demographics, and past interactions with the system (via the feedback attributes). The latter were added so that repeated messages could be used or avoided, if necessary, as determined by the learning algorithm.

Let x_i,t_ denote a vector of the attributes above, for person i at time t, and let y_i,t_ denote the change in activity from day t to day t+1, that is, the number of minutes of activity on day t+1 divided by those on day t. Following the Kesler construction [20], we augment x_i,t_ with an action vector A such that the j-th element of A is equal to 1 if and only if message j was sent on day t.

The training data consisted of all previously collected x_i,t_ and y_i,t_ pairs. We trained a learning algorithm, specifically a linear regression algorithm with interactions, to predict y_i,t_ from x_i,t._

The learning algorithm was rerun every day, and the most up-to-date model was used for prediction.

To predict the most appropriate action on day ρ, we applied the model to each x_i,ρ_ and computed the resulting predicted y_i,ρ_. We then performed Bolzmann sampling [20] with T_Boltzmann_=5 on the outputs of the learning algorithm to choose the feedback message to be given. Thus, actions were chosen relative to their predicted effectiveness. This was done so that actions predicted not to be the best ones would still be tested, in addition to exploiting those actions predicted to be the best ones for the user. T_Boltzmann_ was set based on the initial policy data so that each of the four messages was sent with a probability higher than 10%. We did not adjust this parameter during the experiment.

**Table 1:** Patient characteristics.

Characteristic	Treatment	Control
Number	20	7
Gender	8 female	1 female
Age in years, mean (SD^a^)	58.7 (2.1)	55.1 (3.6)
Initial HbA1c^b^ (%)	7.7	8.7

^a^SD: standard deviation.

^b^HbA1c: glycated hemoglobin.

There are many algorithms for addressing reinforcement learning problems. Most algorithms (Q-learning, TD learning, etc) rely on either having access to the true underlying state, or to high-quality features that represent the dynamics well. In our approach, we mostly tried to predict the effect of different actions on the immediate activity level given the current state of the patient rather than trying to change the patient’s state. Thus, our policy is more of a “contextual bandit” type of algorithm [12]. Although we believe that introducing a state could be immensely useful, having statistical validity to it seems to require amounts of data beyond what we can expect.

## Results

We report the characteristics of patients and their participation in the experiment, the effect of individual messages on user behavior, how the algorithm changed over time, and finally the two experimental outcomes—the change in activity made by participants during the experiment and the change in HbA1c during the experiment.

### Patient Characteristics

A total of 27 patients were recruited, successfully installed the mobile app, and transmitted data for at least 1 week. Patient characteristics are shown in Table 1.

### App Use and Physical Activity Measured

Target physical activity was on average 139 min (standard deviation [SD] 62) per week. The app continued to provide activity data for an average of 20 weeks (SEM 1.6). No statistically significant difference was observed between the treatment and control arms (ranksum, *P*=.30). Interrupted transmission was mostly because of the change of the mobile phone or phone number during the study. Analysis was done on all participants who successfully initiated app use, including the participants who did not complete the 26 weeks of the experiment. Thus, dropout was relatively late in the experiment.

All patients reported that they did not perform regular physical activity before recruitment, but there is naturally no objective accelerometer data for the amount of activity performed before recruitment. We decided that we could not separate the timing of providing the physical activity prescription from the recruitment process without causing any data collected in the first few weeks after recruitment to be biased.

### Effect of Different Messages Over Time

We explored how each of the messages separately and how each two consecutive messages affected the change in the amount of activity and found significant differences in the reaction of participants to different messages and message sequences.

**Figure 2:**
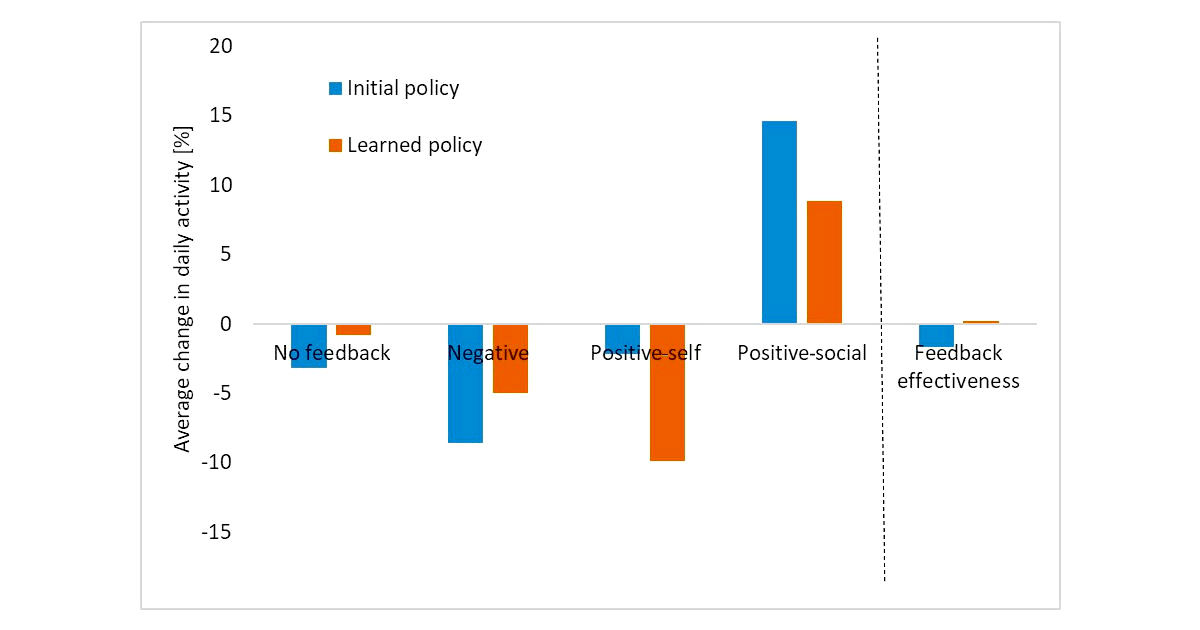
Change in activity following feedback messages for the two feedback policies. Shown are the average improvement in activity for each of the four messages, as well as the feedback effectiveness, which is the improvement in activity weighted by the probability of each message.

Figure 2 shows the average improvement in activity (y_i,t_) for each of the four messages and the feedback effectiveness, which is the improvement in activity, weighted by the probability of each feedback message. The best increase in activity was found on the day after a positive-social message, whereas negative messages and positive-self messages led to a decrease in the amount of activity. This is congruent with experiments conducted by Elliot and Church [18]. The differences in the change of activity between the initial policy and the learned policy were statistically significant (analysis of variance, ANOVA; *P*=.004).

**Figure 3:**
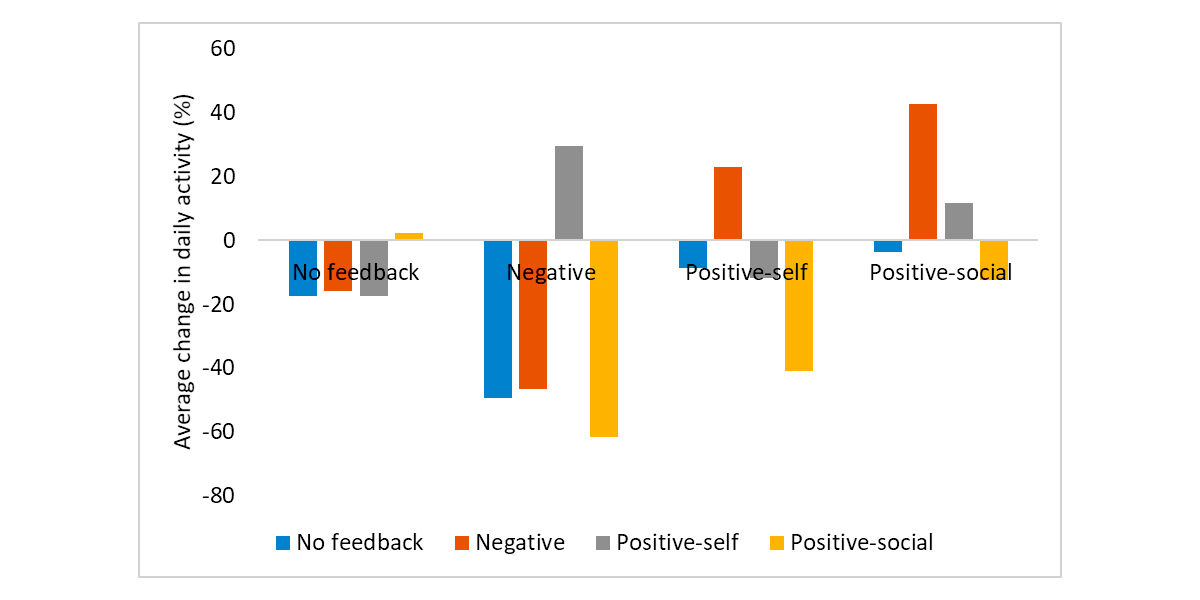
Change in activity as a function of feedback, grouped by current feedback. Each group shows the average change in activity following the current feedback (eg, feedback at time t), given the previous feedback given to the user (at time t-1).

One of the attributes given as inputs to the learned policy was the time since each feedback message was sent. This provides a limited form of historical context to the policy, allowing feedback to be dependent between days. Figure 3 shows the average improvement in activity for feedback on day N, given the feedback on the previous day (N-1). We note that this figure is based on pairs of messages chosen by the algorithm and not random selection.

As the figure shows, for example, even though on average negative feedback produces a negative change in activity, negative feedback is correlated with a positive change in activity if given before a positive-self feedback. Similarly, positive-social feedback repeated day after day is correlated with a lower change in activity.

Differences in activity were statistically significant (ANOVA; *P*=.059 for the previous action and *P*=.02 for the current action, and *P*=.02 for the interaction of the two actions). Thus, time-dependent feedback is correlated with higher average change in activity.

**Variability in Patient Response**

The average improvement in activity varies among patients. To demonstrate this, we represented each user according to the average change in their activity following each daily feedback message (ie, a four-dimensional vector). We limit this analysis to 22 users in the treatment arm who received at least two different messages.

**Figure 4:**
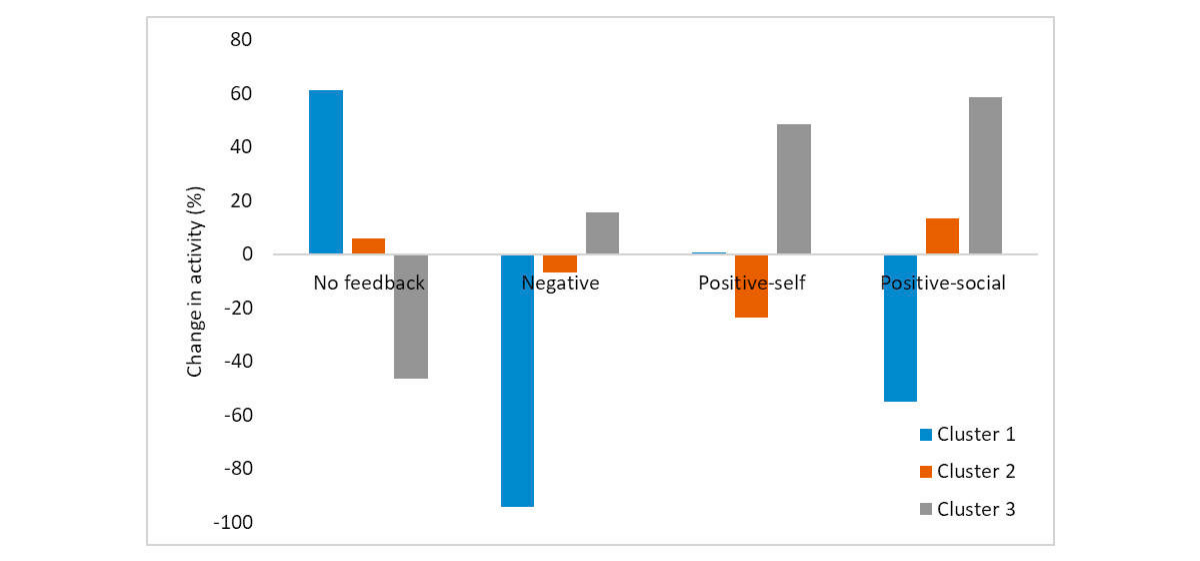
Change in activity as a function of feedback message in each cluster. Cluster 1 comprised 4 patients, cluster 2 had 9 patients, and cluster 3 had 5 patients.

Figure 4 shows the results of clustering users using k-means with 3 clusters. As the figure demonstrates, one group of patients (cluster 1) reacted negatively to any feedback message. In contrast, patients in cluster 3 reacted positively to messages, especially positive-social or positive-self. This demonstrates the importance of individually tailored feedback delivered by our algorithm.

Users in the different clusters differed in their demographics. Table 2 shows the percentage of females and the average age of patients in each cluster. As the Table shows, cluster 3, where patients reacted positively to messages (Figure 4), is dominated by males. In contrast, cluster 2, where reactions to messages were overall weaker, comprises mostly women. Age variations are minor across clusters. Indeed, an ANOVA model with age and gender as independent parameters shows that age is not statistically significantly correlated with change in activity, whereas gender is (*F*=9.65, *P*=.002). Thus, there are significant correlates between patient gender and reaction to messages, demonstrating the importance of tailoring feedback according to these parameters and therefore providing them to the decision algorithm.

**Table 2:** Demographics of patients by cluster.

Demographic	Cluster 1	Cluster 2	Cluster 3
Female (%)	50 (2/4)	67 (6/9)	20 (1/5)
Average age, in years	57	54	56

### The Learning Process of the Algorithm Over Time

**Figure 5:**
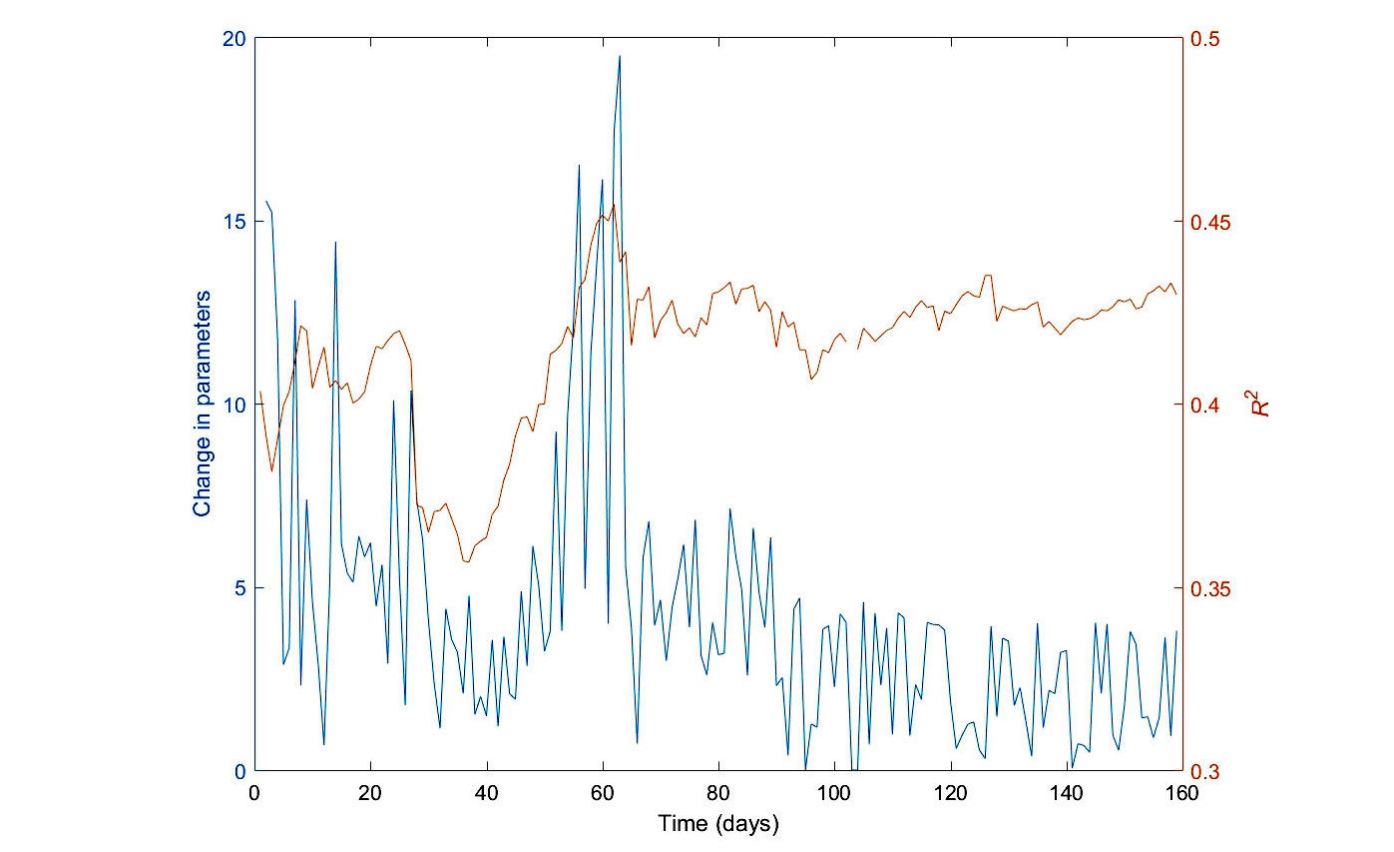
Learning algorithm stability (change in parameters) and predictiveness over time. The horizontal axis is time as the learning algorithm was applied to the experiment. The left vertical axis and the blue lines denoted by plus signs shows the change in algorithm parameters from day to day, and the right vertical axis and full brown line shows the R^2^ value of the model.

We investigated how the messages generated by the learning algorithm changed over time, as more information was collected on the response of the participants to feedback vis-a-vis their previous activity and demographics. Figure 5 shows how the learning algorithm gradually improves over time in predicting the amount of activity, demonstrating that much of the difference in exercise on a given day can be explained by the learning algorithm, which in turn indicates that much of patient behavior is predictable.

Figure 5 shows the change in parameters of the algorithm from one day to the next, calculated as the difference between the absolute values of the model parameters over successive days, and how much of the activity is explained by the predictions of the learning algorithm, as given by the adjusted *R*^2^, over time.

First, we note that stability increases over time, as more data are collected. Second, *R*^2^ initially increases, reaching approximately 0.43. This means that much of the difference in exercise on a given day can be explained by the learning algorithm attribute, indicating that to a large extent, patient behavior is predictable. We also note jumps in learning algorithm stability, for example, around day 60. These jumps seem to correspond to major adverse weather events and may be caused by new ways in which people behave because of these events, creating unexpected data that cause the algorithm to learn a new hypothesis. This demonstrates the necessity to collect longitudinal data over wide-ranging circumstances and possibly the need to include other variables such as weather and calendar events.

We analyzed the coefficients of the learned model to find the coefficients that affect the predictive ability of the learning algorithm. The coefficients that had statistically significant values (*P*<.05) in the linear model were as follows:

1. The interactions between daily activity in the day before feedback is given and
  - the feedback message to provide.
  - the activity performed so far.
  - the time since each feedback message was given.
2. The interactions between the activity performed so far and the time since each feedback message was given.
3. The interactions between the fraction of activity performed so far and the time since each feedback message was given.
4. The interactions between the time since each feedback message was given.

### Improvement in Activity Quantity and Walking Rate

**Figure 6:**
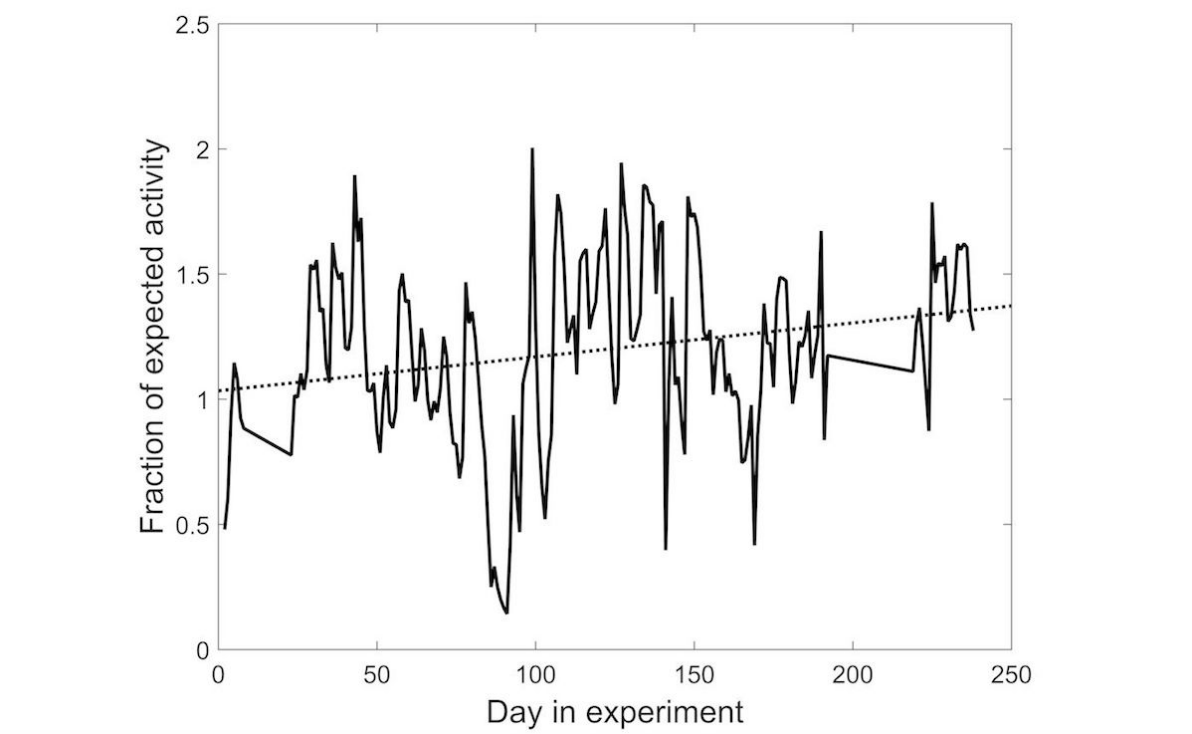
The change in activity (shown as the fraction of the expected activity) over time for one sample user. The dotted line shows the linear slope of the curve.

We modeled the change in activity performed by patients over time (presented as fraction of target activity) using linear regression. Figure 6 shows an example of the fraction of expected activity performed by one participant, together with the linear slope (which, for this patient, is equal to 0.0016) of this activity over the duration of the experiment.

A linear function was fit for each participant separately, and the average slope for the participants in each policy group (weighted by the fit of the linear function) is shown in Table 3. As the table shows, the slope of the learned policy was superior to both the control population and the initial policy. Whereas the latter two show a negative change in activity, the learned policy shows a positive slope, implying in increase in activity over time.

**Table 3:** Rates of improvement in physical activity performed and in the rate of walking. The standard error of the mean is shown in parenthesis. The slope of change in activity is measured by a linear fit to the plotted amount of daily exercise over time. The slope of the rate of walking is the change in the number of steps per minute during walking over time.

Characteristic	Treatment		Control
	Initial	Learned	
Change in activity (minutes walking/day)	−0.001 (0.008)	+0.012 (0.002)	−0.004 (0.002)
Change in rate of walking (Hz/day)	−0.009 (0.005)	0.002 (0.005)	−0.010 (0.007)

The rate of walking (steps per minute) was measured throughout the experiment. We modeled the change in the average weekly rate of walking over time using a linear model by fitting a linear function to the rate of walking for each participant separately over time, and the average slope for the participants in each policy group (weighted by the fit of the linear function) is shown in Table 3. Patients in the control condition reduced their walking rate as the experiment progressed, consistent with the amount of walking they performed. In contrast, the personalized message population increased their walking rate over time significantly.

### Change in Glycemic Control

The initial HbA1c for all participants was 7.8 % (SD 1.0), and, on average, there was an improvement of 0.28 % (SD 0.84) in HbA1c for all patients. As mentioned, intensification of dietary or medical treatment was not restricted, so the change in HbA1c reflects a combination of the change in medical and dietary treatment and the change in exercise.

To assess the effect of variables of participation in the study on glycemic control, we constructed a linear model where the dependent variable is the difference between HbA1c levels at recruitment and the latest available measure of HbA1c. The independent variables are the number of days between measurements, initial HbA1c, and the activity target. Allocation to the personalized policy, higher initial HbA1c, and lower activity targets led to a superior reduction in HbA1c (*R*^2^=0.405, *P*<.01).

**Figure 7:**
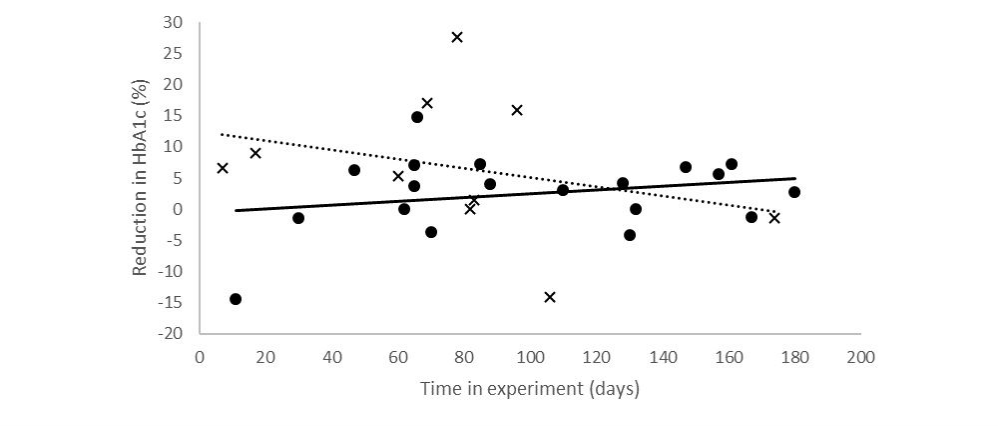
Relative reduction in glycated hemoglobin (HbA1c) over time. Dots represent measurements from people allocated to the personalized policy, whereas crosses represent the control policy. The dotted line is a linear fit to the control policy data and the full line to the personalized policy.

Let HbA1c[t] be the blood glucose measure at time t. The relative reduction in HbA1c is given by (HbA1c[0] – HbA1c[t]) / HbA1c[0] where the beginning of the experiment is at t=0. The relative reduction as a function of the time in the experiment can be seen in Figure 7. The slope of a linear model for the treatment population is positive (0.05, *R*^2^=0.07), whereas that of the control population is negative (−0.06, *R*^2^=0.03), indicating that people in the treatment population experienced a reduction in blood glucose level the longer they participated and received messages determined by the personalized policy.

Thus, we conclude that receiving personal messages is associated with a statistically significant reduction in HbA1c levels. We note, however, that HbA1c values are known to suffer from significant intrasubject variability [21], and thus, future work will require larger cohorts to validate these findings.

### Participant Satisfaction

The results of the patient satisfaction questionnaire are shown in Table 4. Interestingly, both control and learned policy group participants reported increasing their physical activity. The learned policy population reported that the SMS messages helped them increase and maintain the level of their activity significantly more than did the control population (*P*<.01). None of the participants in the control constant weekly reminder group felt that the SMS messages were helpful. Both groups said they received enough messages, though we interpret this result for the control population as unanimous lack of satisfaction with the unchanging wording of the feedback they received.

**Table 4:** Results of the patient satisfaction questionnaire. Only the response to the second question is statistically significantly different between control and personalized messages (chi-square test).

Question	Fraction answering “yes”		*P* value
	Treatment	Control	
Did you increase your level of physical activity since joining the experiment?	0.56	0.67	.73
Did the SMS^a^ messages help you increase the frequency of physical activity?	0.80	0.00	.01
Did the SMS messages help you maintain your physical activity?	0.88	0.33	.07
Do you think you received enough messages to improve your activity?	0.78	1.00	.46

^a^SMS: short message service.

## Discussion

### Principal Findings

A large majority of patients with diabetes are resistant to the usual oral or written recommendations for physical activity they receive when encountering caregivers. Here, we developed a system that takes advantage of the continuous monitoring and communication afforded by smartphones to explore an alternative approach for improving adherence. In this pilot study, we evaluated the effect of feedback messages provided to patients directly by a mobile phone based on their success in obtaining physical activity goals, as measured by a computerized mobile app. This requires careful integration of hardware, software, and human guidance.

Our system used reinforcement learning to learn the feedback that will be most effective for each individual in any given situation, thus creating a highly personalized reminder service. Our results, as evident in the clusters of reactions to different feedback and the effect of message sequences, show the importance of tailoring messages to each individual and at each time.

We found that constant unvarying weekly reminders to perform physical activity are not effective in increasing activity and that patients were not satisfied with receiving them. On the other hand, changing the messages based on the activity performed as determined by the learning algorithm was effective in increasing both the length of time walked and the rate of walking. Indeed, the RL algorithm learned to sequence messages to improve efficiency.

### Strengths and Limitations

In our approach, we learned a single model rather than a plurality of models. We ignored pertinent issues such as the sex and age of the user. It stands to reason that building multiple models from data (eg, one for women and one for men) could yield better results. Such an approach would require a larger population and would probably call for a different type of algorithm that takes into account contextual parameters as well to lead to much better performance [22].

Our approach is fairly unique in that we conducted training within an experiment. In RL terminology, this is called on-policy learning. In many treatments, one must follow an off-policy scheme: collect data using one policy (usually a historical policy) and try to learn a new policy without actually executing it. This leads to several problems such as large variance and bias, as exploration cannot be done where it matters most [23]. In our setting, this was not the case, and we had the luxury of training and using the same policy.

Patients were satisfied with the experience of using the app when they received personalized messages generated by the algorithm. The length of participation and allocation to the learned policy group for which the learning algorithm was used were correlated with superior improvement in HbA1c over competing policies, namely, weekly reminders and policies that do not take into account the specific context and attributes of each user.

Our results suggest that this novel concept for increasing physical activity can be implemented economically, efficiently, and effectively, leading to desired highly positive results. Notice that our approach not only allows for a predictive tool (going beyond current messaging systems) but also provides a method for personalized care.

The use of mobile phones as measurement tools is advantageous in that it does not require patients to maintain a separate device to participate in the experiment. It also has drawbacks in that measurements may be less accurate than those of dedicated devices and that patients may sometimes forget to carry them during exercise, leading to an underestimate of their exercise levels.

### Conclusions

This small-scale study demonstrates the general concept that continuous monitoring and personalized guidance generated by a computer can have a significant impact on patient behavior. Unlike many current e-medicine systems that require input from the patient or the health care provider, the use of an automatic algorithm can be applied to very large groups of subjects. We plan to expand our result to the even more general concept that digitally generated health coaching of humans can have a positive impact. Further studies at larger scale and for longer periods are needed to evaluate whether the digital revolution and the potential to directly communicate with large groups of subjects and to assess the actual behavior reinforced can lead to a major improvement in their health-related behaviors or in their actual health.

## Abbreviations

ANOVA: analysis of variance
HbA1c: glycated hemoglobin
JITAI: just-in-time adaptive interventions
RL: reinforcement learning
SD: standard deviation
SMS: short message service
